# The Use of Salt in the Hygiene and Therapeutics of the Skin

**Published:** 1888-03

**Authors:** H. G. Piffard

**Affiliations:** New York


### THE USE OF SALT IN THE HYGIENE AND THERAPEUTICS OF THE SKIN.

BY H. G. PIFFARD, M. D., OF NEW YORK.

Ordinary sea water contains two per cent. of saline matter. It also contains an organic matter which gives to the skin a slimy feel after coming out of the bath. On entering sea water at a temperature of 70° a momentary chill is experienced, which soon passes off. In the course of half an hour a second chill is felt, due to the gradual abstraction of the body heat. This persists as long as the individual remains in the water. In robust individuals, who leave the water before the occurrence of the second chill, sea bathing may prove beneficial, but in feeble individuals, the sea bath, as ordinarily taken, is apt to result in harm, mainly, he thought, through abstraction of the body heat by the water. The local effects upon the skin coincide with the general effects. In vigorous individuals psoriasis and chronic eczema will often be benefitted by a short bath followed by rubbing. If the patient be feeble or the bath prolonged, the result will be unfavorable. Prickly heat, pruritic affections and furuncles are often benefitted by sea bathing. The principal precautions with reference to sea-bathing are, not to go into the water when it is too cold, and not to remain in until the occurrence of the second chill.

The author has also experimented with artificial brine, varying in strength fro[illegible] 5 per cent. to 25 per cent. When the percentage reaches 25 per cent., the change can be detected by the feeling. If genuine sea salt is used, the sticky, clammy feeling is apparent. If white salt is employed, there is a sensation of extreme cleanliness. A 5 per cent. solution used as a bath at a temperature of 95 per cent., and the immersion continued fifteen to twenty minutes, removes the bodily odors and exudations better than a bath with soap, and the body remains free from odor for a longer period than if soap had been used. The skin presents a condition of softness not seen after any other bath. A bath of a 20 per cent. to 25 per cent. salt solution had been also used with no irritating effect except upon some mucous membrane. In this strength of solution, the water does not seem to wet the skin, but rolls off, leaving the body dry.

In acute eczema the use of water is, as a rule, followed by a temporary aggravation of the trouble. In these cases a full bath of 0.5 per cent. to 1 per cent. solution had been used with great comfort to the patient. In subacute eczema, psoriasis, furuncles, in irritable summer rash, whether papular or pustular, and in ulcerating syphilides, a 5 per cent. solution of salt may be used as hot as can be borne, the bath continued fifteen to twenty minutes, and should be taken just before retiring. Genuine sea salt is not so good for the bath as coarse white salt, on account of the slimy feeling that is left. The therarpeutic effects are identical.

